# Liquid Shear Exfoliation of MoS_2_: Preparation, Characterization, and NO_2_-Sensing Properties

**DOI:** 10.3390/nano13182502

**Published:** 2023-09-05

**Authors:** Pingping Ni, Mbaye Dieng, Jean-Charles Vanel, Ileana Florea, Fatima Zahra Bouanis, Abderrahim Yassar

**Affiliations:** 1 LPICM, CNRS, Ecole Polytechnique, Institut Polytechnique de Paris, 91128 Palaiseau, France; pingping.ni@polytechnique.edu (P.N.); mbaye.dieng@polytechnique.edu (M.D.); jean-charles.vanel@polytechnique.edu (J.-C.V.); 2 COSYS-IMSE, University Gustave Eiffel, F-77454 Marne-la-Vallée, France; 3 CRHEA, CNRS, Université Cote d’Azur, UMR7073, Rue Bernard Grégory, 06905 Sophia-Antipolis CEDEX, France; if@crhea.cnrs.fr

**Keywords:** MoS_2_ nanosheets, liquid shear exfoliation, vacuum-assisted filtration, NO_2_ chemiresistive sensors

## Abstract

2D materials possess great potential to serve as gas-sensing materials due to their large, specific surface areas and strong surface activities. Among this family, transition metal chalcogenide materials exhibit different properties and are promising candidates for a wide range of applications, including sensors, photodetectors, energy conversion, and energy storage. Herein, a high-shear mixing method has been used to produce multilayered MoS_2_ nanosheet dispersions. MoS_2_ thin films were manufactured by vacuum-assisted filtration. The structural morphology of MoS_2_ was studied using ς-potential, UV–visible, scanning electron microscopy (SEM), atomic force microscopy (AFM), energy-dispersive X-ray spectroscopy (EDX), transmission electron microscopy (TEM), X-ray diffraction (XRD), and Raman spectroscopy (RS). The spectroscopic and microscopic analyses confirm the formation of a high-crystalline MoS_2_ thin film with good inter-sheet connectivity and relative thickness uniformity. The thickness of the MoS_2_ layer is measured to be approximately 250 nm, with a nanosheet size of 120 nm ± 40 nm and a number of layers between 6 and 9 layers. Moreover, the electrical characteristics clearly showed that the MoS_2_ thin film exhibits good conductivity and a linear I–V curve response, indicating good ohmic contact between the MoS_2_ film and the electrodes. As an example of applicability, we fabricated chemiresistive sensor devices with a MoS_2_ film as a sensing layer. The performance of the MoS_2_-chemiresistive sensor for NO_2_ was assessed by being exposed to different concentrations of NO_2_ (1 ppm to 10 ppm). This sensor shows a sensibility to low concentrations of 1 ppm, with a response time of 114 s and a recovery time of 420 s. The effect of thin-film thickness and operating temperatures on sensor response was studied. The results show that thinner film exhibits a higher response to NO_2_; the response decreases as the working temperature increases.

## 1. Introduction

During the previous decade, different sensing materials have been studied to develop high-performance gas sensors [1,2,3]. Among them, two-dimensional transition metal dichalcogenides (TMDs) nanostructured materials have gained attention as promising candidates for gas-sensing applications due to their unique electronic, optical, and mechanical properties [4]. In particular, MoS_2_ nanomaterials have demonstrated potential applications for integrated flexible electronics [5] and gas sensing [6], with superior gas sensitivity and selectivity [7]. In the past decade, various types of MoS_2_ nanostructures, including nanosheets, quantum dots, flowers, and nano-meshes, have been fabricated [8]. Among these morphologies, nanosheet structures have received particular attention due to their facile preparation method, highly exposed active sites, and relatively higher specific surface area per amount of material, which is superior for sensing applications [9]. 

Recent years have witnessed increasing research activity on using TMD nanosheets as an excellent sensing material for the fabrication of wearable sensing devices due to their high carrier mobility, mechanical strength, and the fact that no cracks form during the transfer from a rigid substrate to a flexible substrate [10]. Efficient exfoliation of single- or few-layered MoS_2_ nanosheets can be achieved by various approaches, including lithium intercalation, sonication, mechanical exfoliation, etc. [11]. The liquid-phase shear exfoliation (LPSE) method is one of the most successful methods for the preparation of TMD nanosheets from the bulk phase of the materials in liquid dispersions, making it attractive to produce functional inks for printable electronic devices and thin films for various applications, including gas sensing. Although great progress has been made in this field, there are still many challenges regarding the availability of TMD inks and the large-scale fabrication requirements of flexible/wearable devices. In particular, the availability of solution-dispersible TMD nanosheets that can be used to deposit, or print, a large-scale thin film on a variety of non-conventional substrates for the fabrication of TMD-based electronic devices [12,13]. While the sensing properties of MoS_2_ nanosheets to various gases have been reported, most of these studies focused on materials mechanically exfoliated [14], CVD-grown [15], and sonication-assisted liquid-phase exfoliation methods [16]. The simplicity of this later method is attractive; however, it has many drawbacks, such as time-consuming, inferior product quality, low yields, and reproducibility. Shear exfoliation is similar to sonication exfoliation and can be regarded as one of the most industrially feasible methods for mass production of functional inks based on defect-free few-layer TMD materials [17]. Besides solution TMD material synthesis, thin-film processing methods that can be used to coat the colloidal dispersion of nanosheets onto a chosen substrate as patterns and functional thin films are highly desirable. Assembly of TMD-material films from the solution phase can be accomplished by various coating techniques, among which the vacuum-assisted filtration method is an easy and effective way to produce 2D-material thin films [18]. So far, thin-film MoS_2_ fabrication has been mainly limited to the drop-casting method. Although this method is useful for coating a very thin layer, it suffers from a few drawbacks, e.g., the fact that the quality in terms of thickness is not uniform, the inhomogeneity of the films, and the coffee ring effect, which may hamper the performance in many applications. A deep understanding of the effect of material production, thin-film processing methods, and device design on the MoS_2_ sensing of NO_2_ is necessary in order to develop a reliable NO_2_ sensor. We reasoned that the development of solution-processed MoS_2_ nanosheets from LPSE would allow greater control over the fabrication of large-area MoS_2_ thin-film nanosheets and their integration into multifunctional devices. To evaluate the feasibility of this approach, we herein present the development of LPSE of MoS_2_ and a vacuum-assisted filtration method to fabricate large-area MoS_2_ thin films for gas sensing applications. Besides production and thin-film processing, a multiscale physico-chemical characterization of the produced nanosheet materials by means of various techniques, including ς-potential, UV–visible, Raman spectroscopy, atomic force (AFM), scanning electron microscopy (SEM), high-resolution transmission microscopies, and X-ray diffraction, coupled with electrical measurements, was conducted to gain a deep understanding of the structure–properties relationship. Finally, the NO_2_-sensing properties of MoS_2_ thin-film nanosheets have been investigated at room temperature in dry nitrogen.

## 2. Materials and Methods

### 2.1. Preparation of MoS_2_ Dispersion

The MoS_2_ water dispersion was prepared by the LPSE method, following the protocol previously reported [19,20]. The process of liquid exfoliation consists of two steps: (i) Pre-treatment and (ii) final exfoliation. In a typical preparation, 4 g of MoS_2_ powder was first immersed in an 80 mL aqueous solution of sodium cholate (NaC) with a concentration of 7 g/L and sonicated for 30 min, followed by shear force mixing using a Silverson L5M-A mixer was applied at maximum speed (10,000 rpm) for 4 h. During the shear, the temperature of the solution was kept constant with an ice bath to prevent the dispersion from overheating. Afterwards, the dispersion was left overnight to settle before the supernatant was discarded. In this way, all of the aqueous-soluble contaminants that are present in MoS_2_ powders are eliminated during the pre-treatment stage. Then, the sediment was re-dispersed in 80 mL of a fresh solution of NaC (concentration: 7 g/L) and exfoliated for a further 6 h at the same rate using an ice bath as in the pre-treatment stage. The top 2/3 of the supernatant was collected after settling overnight and centrifuged for two hours at 1500 rpm to remove unexfoliated MoS_2_. The supernatant after centrifugation was collected for further analysis, while the sediment was discarded.

### 2.2. MoS_2_ Thin-Film Fabrication

To avoid aggregation, the MoS_2_ solution was sonicated for 30 min to obtain a uniformly dispersed MoS_2_ solution before each use. Then the MoS_2_ dispersion was diluted in 100 mL of water and subjected to sonication for an additional 30 min to help MoS_2_ nanosheets homogenously disperse in water. The MoS_2_ films were fabricated using vacuum-assisted filtration on a nitrocellulose membrane, an MF-Millipore^®^ (Merck, Molsheim, France), with a pore size of 25 nm. The self-assembly films were then dried in a vacuum overnight. The self-assembly MoS_2_ film on the membrane was then cut into sizes of choice and pressed against a cleaned substrate surface, which had previously been wetted with several drops of isopropanol, with the MoS_2_ side in contact with the substrate. To ensure that the membrane was firmly attached to the substrate, we pressed it to remove the air at the interface. Finally, the membrane was dissolved using an acetone solution overnight, followed by a thorough cleaning with sufficient acetone and ethanol to remove the membrane’s residue.

### 2.3. Characterization Methods

UV–visible absorption spectra were carried out on an Agilent Technologies Cary 60 UV–vis spectrophotometer. Dynamic light scattering (DLS) and zeta potential measurements were performed with a Malvern Zetasizer Nano. Scanning electron microscopy (SEM, Hitachi S4800 microscope, Tokyo, Japan) was used to investigate the microstructure of MoS_2_ film. The chemical state of the synthesized materials was analyzed using energy dispersion spectroscopy (EDS) (Thermo Ultra Dry-Electron Corporation, Waltham, MA, USA). The surface roughness of the MoS_2_ thin film was obtained by using a Dimension Icon instrument from Bruker with the scan A system, which operated in tapping mode using a standard AFM probe featuring a pyramidal silicon tip (325 kHz, 40 N/m). X-ray diffraction measurements were performed on a Bruker D8 Discover (Germany) diffractometer using a Cu Kα source (λ1 = 1.5406 A, λ2 = 1.5444 A) and 2θ scans from 5 to 80°. Raman spectra were recorded using a high-resolution confocal Raman microscope (Labram HR800; HORIBA Jobin Yvon, Palaiseau, France) with a microscope lens of 100 (NA = 1). Micro-Raman mapping was performed in high-resolution mode using a laser excitation of λ = 532 nm with a 20 s scan time and two accumulations per spectrum. Transmission electron microscopy (TEM) (Titan-Themis Thermo Fisher electron microscope operating at 80 and 300 kV) was performed to investigate the quality of the prepared films. 

### 2.4. Sensor Design, Fabrication and Electrical Performances

**Figure 1:**
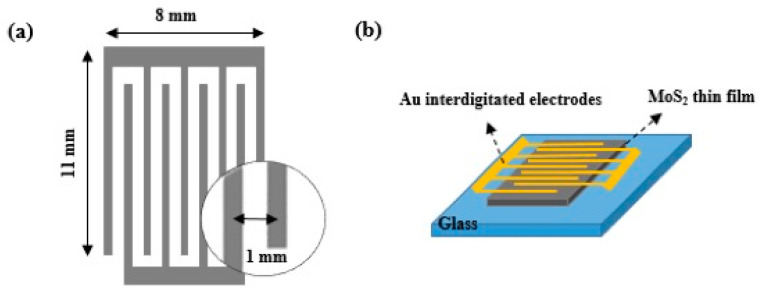
Schematic illustration of (**a**) interdigitated electrodes and (**b**) sensing device.

For gas sensor tests, the interdigitated electrodes were fabricated on top of 300 nm-thick MoS_2_ films transferred on glass substrates via the thermal deposition of 100 nm-thick gold through a metal shadow mask. The size of the interdigitated electrode is 11 mm × 8 mm. The interspacing between the adjacent fingers is typically 1 mm, with the width of interdigitated electrodes being 0.2 mm, as shown in Figure 1a. The fabricated MoS_2_ sensors were annealed for 2 h at 100 °C in N_2_ at ambient pressure to desorb attached gaseous molecules on the surface and then loaded into a sensing chamber as described in our previous work [20]. The electrical measurements were performed using a semiconductor parametric Keithley 4200-SCS (Tektronix company, Les Ulis, France) analyzer under ambient conditions. The measurements were carried out by applying a scan rate of 10 mV s^−1^.

### 2.5. Gas Sensing Measurements 

The gas sensing measurement system consists of a gas generator (V-OVG) (Owlstone, Westport, CT, USA), a Nextron chamber with micro-probe system analysis, a humidity control system (1–97% relative humidity (RH) and 0.01 resolution), and a temperature control system (20–200 °C). The concentration of NO_2_ is controlled by a gas generator using permeation tube technology. An NO_2_ permeation rate of 6025 ng/min at 30 °C was used. Dry N_2_ (99.99%) was used as the dilute gas to reach the desired concentration. The working temperature of the fabricated sensor was controlled with a commercial Nextron control system (−20–300 °C) connected to a computer. All sensing tests were carried out at room temperature with a relative humidity of 30%. Residual gas was removed by purging the chamber with N_2_ flow for 30 min prior to introducing the gas. Before sensing measurements, the sample was annealed at 100 °C in order to desorb all volatile molecules and residues on the surface of MoS_2_. The response of the sensor upon exposure to NO_2_ is defined as:

(1) $$S_{\mathrm{resp}}\;(\%)=\frac{R-R_{0}}{R_{0}}\times 100$$

where S_resp_ is the sensitivity (response) of the gas sensors, and R and R_0_ are the resistance of the sensors after and before exposure to NO_2_ gas, respectively.

## 3. Results

### 3.1. Characterization of MoS_2_ Nanosheet Dispersions

#### 3.1.1. UV–Visible Spectroscopy

**Figure 2:**
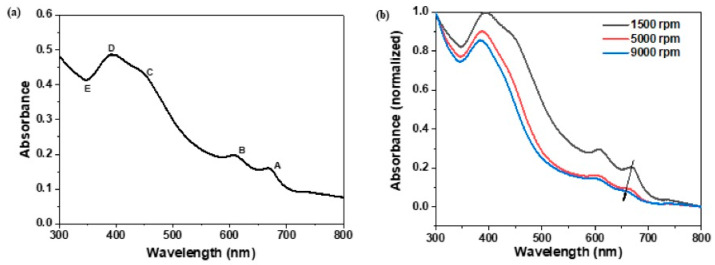
(**a**) UV–visible spectroscopy of MoS_2_ dispersion (after 60 times’ dilution); (**b**) UV–visible spectroscopy of MoS_2_ dispersion after different centrifugation speeds.

MoS_2_ exfoliation was carried out in the two-step protocol following the previous report [19]. The bulk MoS_2_ powders were first mixed together with an aqueous solution of sodium cholate to get a comparable surface energy of 75 mJ/m^2^ [21]. Following, the second step was to exfoliate MoS_2_ material through a high shear process, 6 h of mixing at 10,000 rpm. The liquid phase exfoliation parameters were varied and optimized to maximize the production of the nanosheets. The main idea was to find out the optimal concentration and stability of MoS_2_ nanosheet dispersion because this would help in subsequent MoS_2_ dispersion, which is a prerequisite for ensuring good film formation. The MoS_2_ dispersion was characterized by UV–visible spectroscopy and the DLS technique to determine the concentration, the average layer number of MoS_2_ nanosheets, the average sheet size, and the stability of the solution. Figure 2a shows a typical UV–vis absorption spectrum of the exfoliated 2H-MoS_2_ nanosheet dispersion. As illustrated in this figure, the UV–visible absorption spectrum shows four characteristic bands A, B, C, and D at 670 nm, 608 nm, 450 nm, and 400 nm, respectively. The absorption peaks A and B are excitonic transitions arising from the direct band gap. The theoretical calculations have shown that the A and B excitonic peaks correspond to the energy band splitting due to spin orbitals at point K between the maximum valence band and the minimum value of the conduction band at the K point in the band diagram zone [22]. The C and D absorption bands are assumed to be related to the direct excitation transition of the M point in the Brillouin zone. Additionally, from the absorption spectrum of the MoS_2_ dispersion, one can also extract useful information on the concentration, length, size, and thickness of the nanosheets. It is known that the lateral size and thickness of the nanosheets can be estimated from UV–visible absorption spectrum [17]. Such behavior is related to edge confinement effects; when the electronic band structure of the layered materials is changed, as a result of edge and confinement effects, the magnitude and position of the excitonic transitions change. So, the changes in the optical absorption spectrum reflect the changes in the size and thickness of the nanosheets. Roughly, the A and B absorption bands are related to the layer number of MoS_2_ nanosheets, while the C and D absorption peaks represent the absorption of MoS_2_ with smaller sizes. The mean length (L) of the MoS_2_ nanosheets is dependent on the ratio of extinction at B-exciton to that of E and can be estimated by the formula [23]:

$$L\left(\mu m\right)=\frac{\frac{3.5{\mathrm{Abs}}_{B}}{{\mathrm{Abs}}_{E}}-0.14}{11.5-\frac{{\mathrm{Abs}}_{B}}{{\mathrm{Abs}}_{E}}}$$

where Abs_B_ and Abs_E_ are the intensities of the B (∼605 nm) band and E (∼345 nm), respectively. A value of the nanosheet size of 138 nm and a number of layers of six were estimated according to the Coleman method [23], which are similar to those reported in the literature [19]. The Beer–Lambert law A/l = Cα was used to calculate the concentration of the dispersion, where A/l is the absorbance per cell length of peak A and α is the absorbance coefficient. A nanosheet concentration of 0.63 g/L was determined using an absorbance coefficient of 1517 L·g^−1^·m^−1^ at 672 nm [24].

Sequential centrifugation was used to separate the nanosheets with varying thickness and lateral size distributions from the same stock solution. Figure 2b shows the blue shift of the A-exciton peak in the UV–visible absorption spectrum with decreasing mean nanosheet thickness. The nanosheet dispersions were obtained by sequential centrifugations with increasing speeds from 1500 to 9000 rpm.

#### 3.1.2. Dynamic Light Scattering

**Figure 3:**
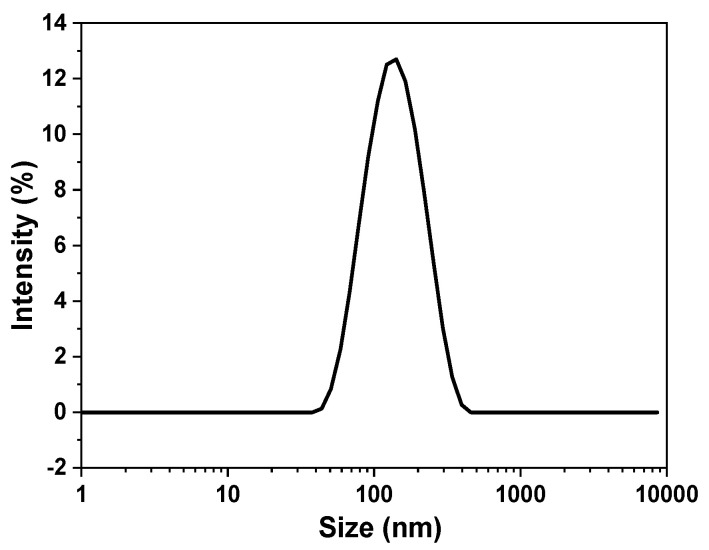
DLS lateral size distribution of MoS_2_ dispersion by intensity.

DLS is a common technique to estimate the mean hydrodynamic size of MoS_2_ nanosheets in solution and is an alternative method to direct TEM or AFM imaging. However, the anisotropy of nanosheet materials renders the use of light scattering more complicated due to their lateral size, thickness, and shape distributions. Coleman et al. [25] characterized MoS_2_ nanosheets with the DLS technique and derived an empirical relationship between the apparent hydrodynamic radius calculated by the Stokes–Einstein relationship and the size estimated from TEM images. Figure 3 shows a typical DLS output graph; the hydrodynamic radius of the dispersed MoS_2_ nanosheets ranges from 44 nm to 340 nm, with a maximum distribution at 142 nm. The mean lateral size deduced from the empirical relationship [25] is 120 nm ± 40 nm, which agrees with the average size of 138 nm estimated from UV–visible absorption measurements. Moreover, the ς-potential, which measures suspension stability, was also determined via DLS. A ς-potential of −39.1 mV was observed, indicating moderate stability of MoS_2_ suspension fabricated using the LPSE method.

#### 3.1.3. Atomic Force Microscopy

**Figure 4:**
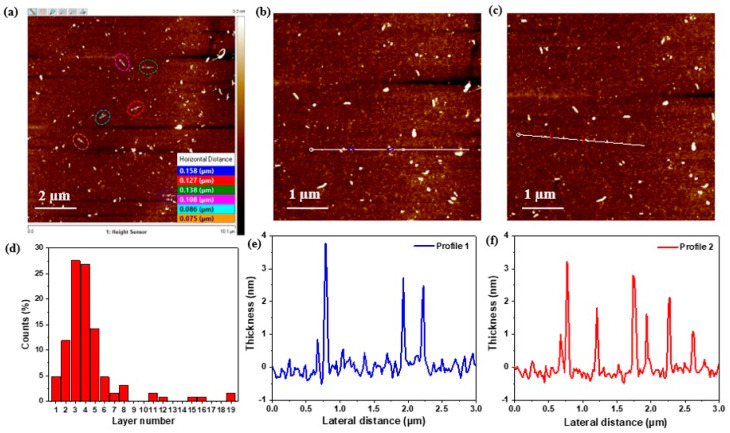
(**a**) Top AFM images of MoS_2_ nanosheets with inset their lateral size profiles; (**b**,**c**) zoomed-in images from image (**a**). Bottom (**d**); layer number distribution of MoS_2_ nanosheets in (**a**); (**e**,**f**) thickness plots of selected MoS_2_ nanosheets in images (**b**,**c**).

AFM was employed to study the sheet thickness and the mean sheet size of the exfoliated NO_2_. To do this, we drop casted the MoS_2_ nanosheet dispersion onto a Si/SiO_2_ wafer. By analyzing the AFM height on a large number of nanosheets and plotting their height profile, the AFM image in Figure 4, along with the layer number distribution graph, shows a clear presence of MoS_2_ nanosheets with a few layers. Most of these nanosheets show the same apparent AFM height of 2–4 nm (Figure 4e,f), with a step height between 0.8 and 1 nm, which agrees with the value of monolayer height of chemically exfoliated MoS_2_ [26]. The number of monolayers per nanosheet of the randomly distributed MoS_2_ nanosheets is in the range of 1–6 monolayers, and this is evident from the corresponding height profiles and statistical analysis plots (Figure 4d–f). The lateral dimensions are in the range of ∼50–150 nm. All these observations are in agreement with UV–visible and DLS results, indicating that a high-speed rotator coupled with a higher shear time is a reliable method to obtain few-layered nanosheets of 2H-MoS_2_ without any severe damage to its final morphology.

#### 3.1.4. High-Resolution Transmission Electron Microscopy

**Figure 5:**
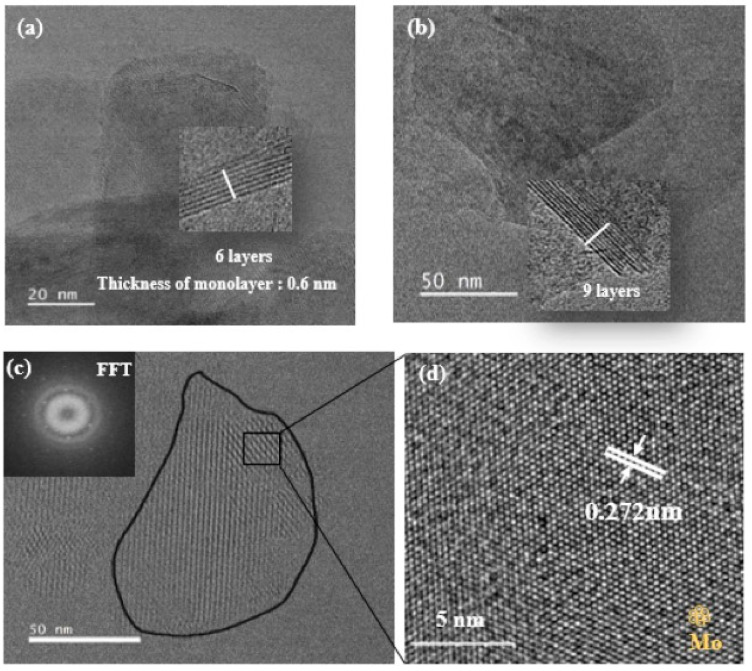
(**a**,**b**) TEM images of 6-layered and 9-layered MoS_2_ nanosheets; (**c**) HRTEM image of MoS_2_ monolayer with inset FFT image; (**d**) HRTEM image of selected area of (**c**).

High-resolution TEM (HRTEM) images were also recorded to estimate the lateral size and number of nanosheet layers of the exfoliated MoS_2_. Figure 5 shows the HRTEM images of MoS_2_ nanosheets, along with their corresponding FFT (fast Fourier transform) results. The HRTEM images provide a nanosheet size of a few hundred nanometers (100–150 nm). The exfoliated sheets consist of 6 to 9 layers, which means that the interlayer distance of MoS_2_ is 0.6 nm (Figure 5a,b). Moreover, Figure 5c presents a larger MoS_2_ grain composed of a small monolayer within the inset of its corresponding FFT of the selected area, illustrating the MoS_2_ hexagonal symmetry in the <001> direction. Figure 5d corresponds to a magnified image of the area represented by the black rectangle in Figure 5c, indicating a perfect crystalline structure with a lattice spacing of 0.272 nm. All these demonstrate that the crystalline structure of the MoS_2_ nanosheets obtained via the LPSE method remains intact after exfoliation.

#### 3.1.5. Raman Spectroscopy

**Figure 6:**
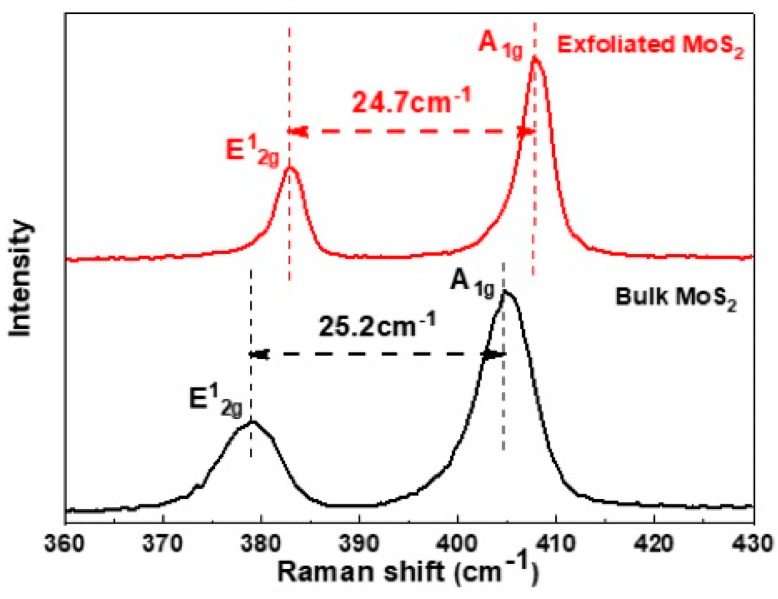
Raman spectra of bulk MoS_2_ (**bottom**) and exfoliated MoS_2_ (**top**).

Raman spectroscopy provides structural and electronic information about TMD materials [27]. The Raman spectra for MoS_2_ obtained using 532 nm excitation are shown in Figure 6. The data were obtained using the minimum power (3 mW) in order to prevent laser heating. Two sets of peaks are visible, corresponding to E^1^_2g_ and A_1g_ modes: For exfoliated MoS_2_, E^1^_2g_ is at 383.05 cm^−1^ and A_1g_ is at 407.71 cm^−1^; for bulk MoS_2_, E^1^_2g_ is at 379.02 cm^−1^ and A_1g_ is at 404.2 cm^−1^, indicating the in-plane and out-of-plane vibrational modes of the MoS_2_ layer. The peak position and the frequency difference (the peak-to-peak separation) of these two modes are sensitive to the layer thickness of MoS_2_. In bulk MoS_2_, the peak-to-peak separation of these modes is 25.2 cm^−1^, while it decreases to 24.7 cm^−1^ in exfoliated MoS_2_ nanosheets, indicating the presence of four to five monolayers [27]. After exfoliation, the A_1g_ and E^1^_2g_ modes shifted towards the higher wavenumber.

### 3.2. MoS_2_ Thin-Film Characterizations

#### 3.2.1. Scanning Electron Microscopy

**Figure 7:**
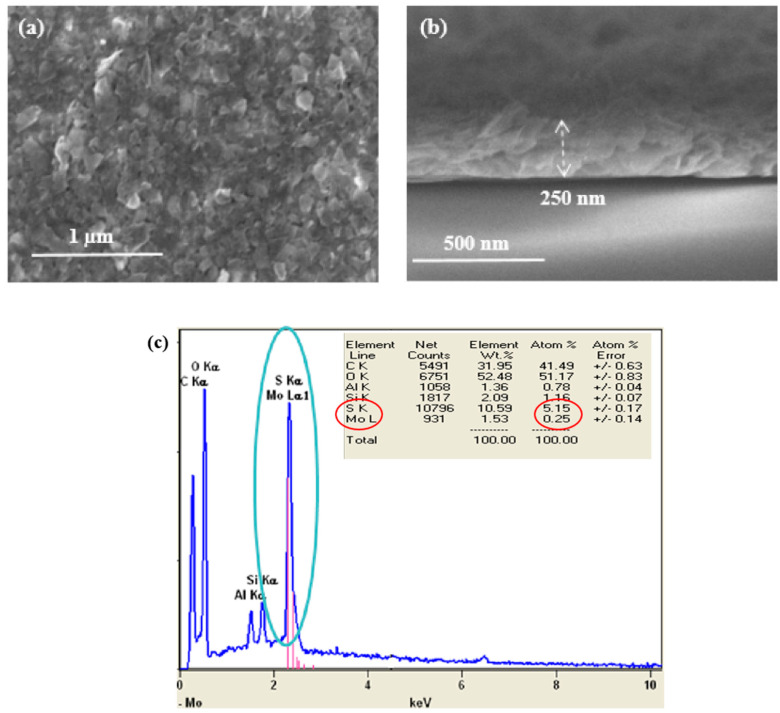
(**a**) Surface SEM image of MoS_2_ nanosheet network fabricated by vacuum filtration process and deposited on ITO glass; (**b**) A cross-sectional image of a 250 nm thick MoS_2_ thin film. (**c**) EDS spectrum of MoS_2_ thin film on Si wafer.

To self-assemble the MoS_2_ nanosheets on the substrate, vacuum-assisted filtration was employed to produce a compact MoS_2_ film from dispersion. For morphological study and electrical characterization, the MoS_2_ film was transferred onto a silicon wafer by dissolving the membrane and washing the obtained MoS_2_ film with acetone. To analyze the surface morphology and film thickness of the MoS_2_ nanosheet thin film, AFM and SEM characterizations were carried out. As shown in Figure 7a, all the films analyzed are tightly packed, and the nanosheets completely cover the substrate without any voids or cracks, forming a continuous porous nanosheet network. As a result, this method produces a film with good inter-sheet connectivity and relative thickness uniformity. The thickness of the MoS_2_ layer is measured to be approximately 250 nm, as can be seen from the cross-sectional view of the SEM image of the MoS_2_ thin film in Figure 7b. To gain more insights into the chemical composition of the exfoliated MoS_2_, energy-dispersive spectrometer (EDS) analyses were carried out. The EDS measurements of MoS_2_ display signals of Mo and S (Figure 7c), which confirm the formation of MoS_2_.

#### 3.2.2. Atomic Force Microscopy

**Figure 8:**
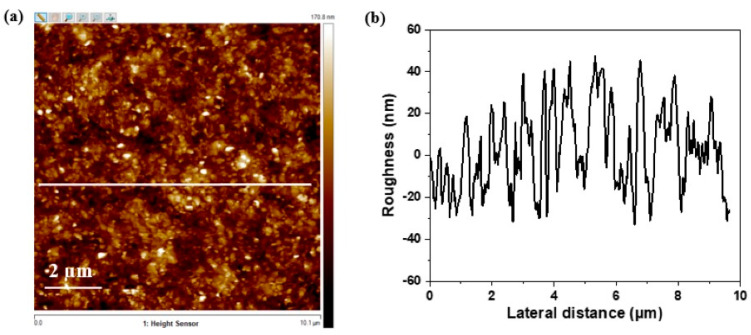
(**a**) AFM image of MoS_2_ thin film on Si wafer; (**b**) roughness profile of the white lines area indicated in image (**a**).

Further, topography analysis of the MoS_2_ thin film was performed by AFM characterization, as shown in Figure 8a. The average roughness Ra of the MoS_2_ thin film was determined using NanoScope Analysis, which is 9.75 nm, revealing a low surface roughness regarding the thickness of the film, 250 nm, and the absence of any appreciable aggregation throughout the filtration process.

#### 3.2.3. X-ray Diffraction

**Figure 9:**
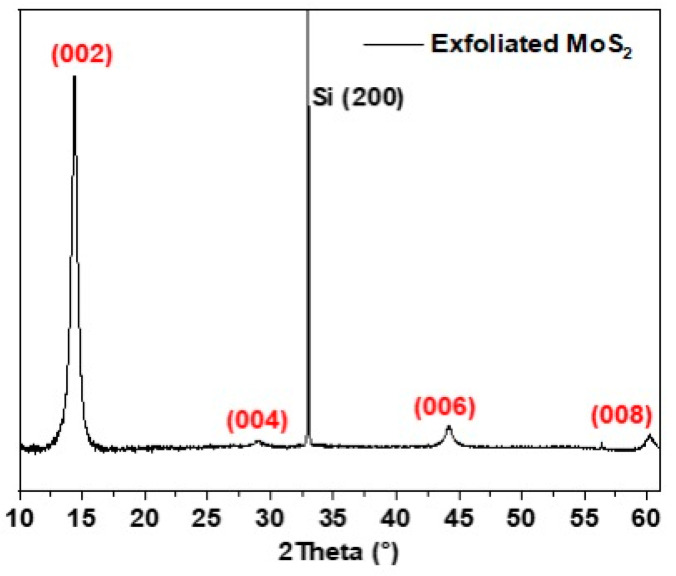
XRD pattern of MoS_2_ thin film deposited on Si (100) substrate.

X-ray diffraction (XRD) was carried out to investigate the phase structure of the exfoliated material. Figure 9 displays the XRD patterns of exfoliated MoS_2_. As can be observed in this XRD pattern, four peaks at 14.38°, 29°, 44.2°, and 61.7° of MoS_2_ are observed, which correspond to the (002), (004), (006), and (008) planes of 2H MoS_2_ (JCPDS:37-1492). The strong and narrow diffraction peak at 14.38° indicates the well-crystallization of MoS_2_ nanosheets. No secondary crystalline phases are observed in the XRD data, indicating that the nanosheets are not oxidized during the exfoliation process.

### 3.3. NO_2_ Sensing Properties

#### 3.3.1. MoS_2_ Based Sensor’s Performance on NO_2_ at Room Temperature

**Figure 10:**
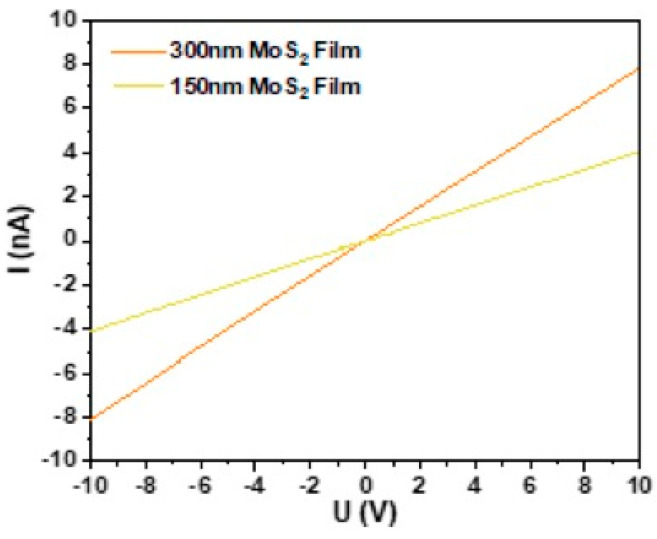
I–V curves of MoS_2_ thin films with different thickness.

MoS_2_ films were transferred onto glass substrates with interdigitated Au electrodes (shown in experimental Section 2.4, Figure 1b). Before gas sensing measurements, the electrical properties of fabricated MoS_2_ thin films were characterized using the two-point I–V method under an applied bias voltage ranging from −10 V to +10 V, as shown in Figure 10. Gold electrodes were selected because it has been reported that gold forms a quasi-Ohmic contact with MoS_2_ monolayers [28]. Firstly, we analyzed the effect of the thickness of the network film on its electrical properties. Two films with different thicknesses were analyzed. The I–V curves for both thinner and thicker films are shown in Figure 10. Both films exhibit good conductivity and a linear I–V curve response, indicating good ohmic contact between the MoS_2_ film and the gold electrodes. This has been attributed to a reduction in the Schottky barrier at the MoS_2_–gold interface for MoS_2_ multilayer nanosheets. In addition, the resistance of thin films decreases from 2.54 GΩ to 1.25 GΩ as the thickness increases, which is a characteristic of the percolation effect in nanosheet networks [29]. Moreover, thicker films have more available charge carriers, which can increase their conductivity. A value of conductivity of 4.02 × 10^−5^ S·m^−1^ was estimated for MoS_2_ nanosheet network film, a value close to that previously reported for MoS_2_ printed films (∼2.5 × 10^−6^ S m^−1^) [29] and solution-processed MoS_2_ films (7 × 10^−7^ S m^−1^) [30].

Having characterized the electrical properties of the MoS_2_ nanosheet network films, we tested these devices for gas-sensing applications. The MoS_2_ chemiresistive sensors were probed inside the test chamber. After being heated at 100 °C for 10 min to remove oxygen and water molecules, the sensors were exposed to a wider range of NO_2_ concentrations (1–10 ppm) for ~5 min at 30 °C. The resistance of the devices was continuously recorded by applying a voltage of 10 V. To check the reproducibility of the analysis, more than four samples were characterized for each concentration. Different areas at the edges and in the central region of the samples were analyzed and typically showed similar characteristics.

**Figure 11:**
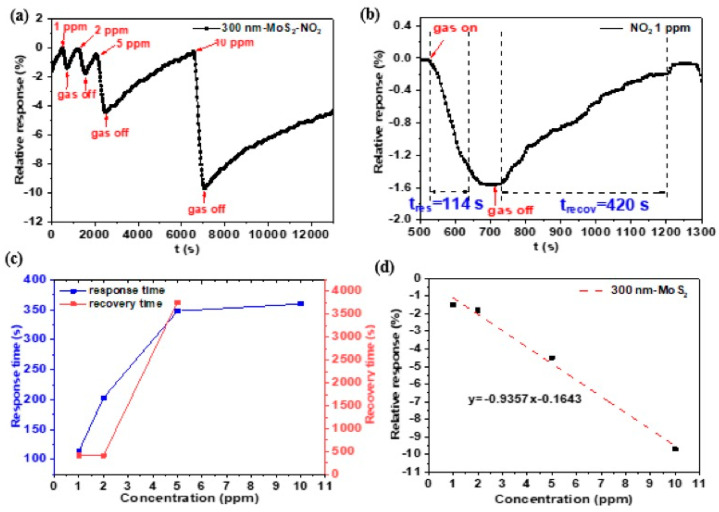
(**a**) Sensing transient of 300 nm thick MoS_2_ thin film to different NO_2_ concentration; (**b**) dynamic response–recovery curves to 1 ppm NO_2_; (**c**) response and recovery time of 300 nm MoS_2_ film at different NO_2_ concentration; (**d**) fitted curve of the sensor response with NO_2_ concentration.

As shown in Figure 11a, the variation of the electrical response of MoS_2_ was found to be 1.5% for 1 ppm and 9.7% for 10 ppm of NO_2_. Figure 11b shows the response and recovery time of MoS_2_ thin film exposed to 1 ppm NO_2_. The fabricated MoS_2_ films show a relatively rapid response time of 114 s to 1 ppm NO_2_, a value comparable to that of a liquid-phase-exfoliated MoS_2_ nanosheet-based sensor [31]. However, the recovery process requires 420 s. Moreover, when exposed to a high concentration of NO_2_, the device takes longer to recover its original resistance. As shown in Figure 11c, the recovery time from exposure to 5 ppm NO_2_ was 1 h, and it took more than 2 h for MoS_2_ thin film to recover from 10 ppm NO_2_. The incomplete recovery at room temperature is due to the strong binding between NO_2_ and the reactive sites of MoS_2_. The long-term recovery phenomenon has been widely reported [32,33] and is known to be due to the high energy binding between the NO_2_ molecules and MoS_2_ surface defects. The sensor shows a linear variation of the response versus the concentration, as seen in Figure 11d, with a sensor response that varies linearly with NO_2_ concentrations ranging from 1–10 ppm with a slope of −0.9357.

As displayed in Table 1, the NO_2_ sensing performance of the shear-exfoliated MoS_2_ films is comparable to those reported in previous works, especially in terms of response, response time, and recovery time.

#### 3.3.2. The Effect of the Film Thickness on NO_2_ Gas Sensing Performance

Some of the recent works on gas sensors based on nanostructured materials have shown a trend of increasing the sensor’s sensitivity when the film thickness decreases, indicating that film thickness is a critical parameter for nanostructured gas sensor performance [34,41]. Table 2 shows the effect of the thickness of the film of the MoS_2_ nanosheet gas sensor on sensitivity. As it is seen from Table 2, two different sensors with thicknesses of 150 and 300 nm were tested. The gas sensitivity of MoS_2_ nanosheet gas sensors increases with a decrease in film thickness. A thinner film was found to exhibit better gas-sensing properties. This finding is in good agreement with previous work that has shown sensors based on rGO–MoS_2_ thin films compared to the thick layer exhibit high sensitivity to NH_3_ at room temperature in the ppm concentration range [34]. Such behavior is commonly observed in metal oxide semiconductor-based gas sensors [41], and it has been investigated by Windischmann and Mark, who showed that the sensitivity of gas sensors based on metal oxide semiconductors is inversely proportional to the carrier concentration [42].

In the case of nanostructured materials, the sensing performance of a thin-film sensor depends on various structural parameters such as the ratio of surface-to-volume, surface roughness, porosity, initial resistance of the film, etc. [43]. Increasing the ratio of surface-to-volume, roughness, and porosity would lead to an increase in the number of adsorption sites and, consequently, the sensing capabilities of the devices. As the thickness of the film decreases, the surface-to-volume ratio of the film increases. As a result, for a thinner sensing layer, the number of sites available for the adsorption of the analyte is expected to be higher, which results in a higher sensitivity.

#### 3.3.3. The Effect of the Working Temperature on Gas Sensitivity

**Figure 12:**
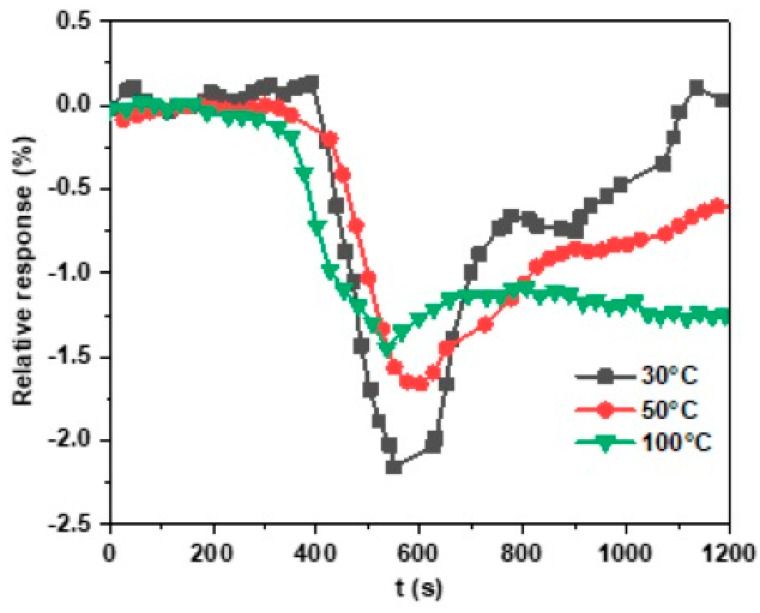
Response of MoS_2_-based sensor to 1 ppm at 30 °C (black line), 50 °C (red line), and 100 °C (green line).

The effect of the working temperature on gas sensitivity was investigated by increasing the working temperature from room temperature (RT) to 100 °C (Figure 12). The response of the MoS_2_ sensor to 1 ppm of NO_2_ was 2.1%, 1.7%, and 1.4% when the temperature increased from 30, 50, and 100 °C, respectively. The high response obtained at RT is attributed to possible sulfur vacancies, as reported for many works on sensors based on TMD materials. Density functional theory calculations have shown that NO_2_ molecules strongly adsorb on edge sites compared to terrace sites in the basal plane of the MoS_2_. Edge sites, as well as defect sites of the MoS_2_, have a larger value of adsorption energy for NO_2_ [44]. Thus, these defects are essential at low temperatures as NO_2_ molecules deplete the material carrier charge through the vacancies and change the electronic conduction [44].

#### 3.3.4. Gas-Sensing Mechanism

**Figure 13:**
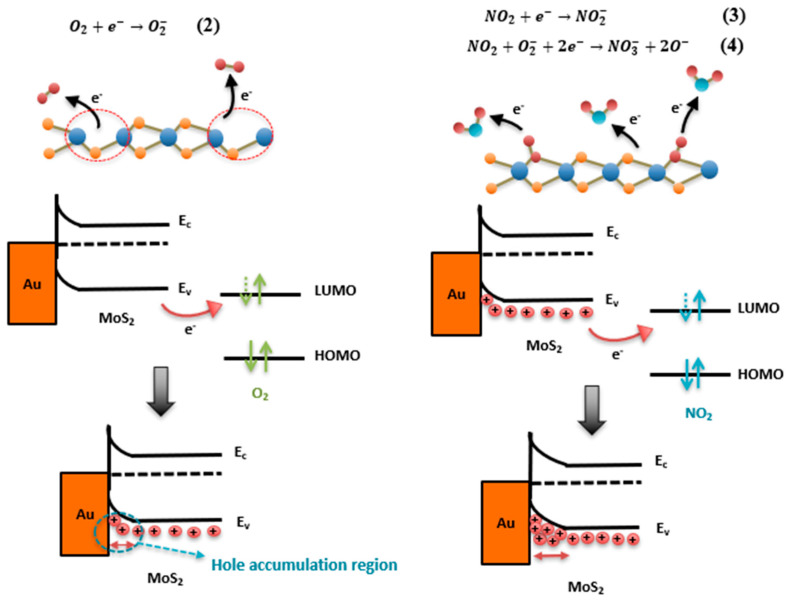
Schematic illustration of NO_2_-sensing mechanism for MoS_2_-based sensors.

Based on previous works on the 2H-MoS_2_ chemiresistive sensor, the sensing mechanism can be explained by molecule adsorption followed by a charge transfer between the sensing layer and the adsorbed gas [41]. The change in the sensor’s resistance is a result of this induced charge. The magnitude of the charge transfer is mainly related to the donor/acceptor characteristics of gas molecules, such as their oxidizing or reducing ability, and the intrinsic electrical properties of the sensing layer, conducting material, or semiconducting material (n- or p-type). In ambient conditions, MoS_2_ is a p-type semiconductor due to the intrinsic S vacancies and oxygen incorporation originating from its exposure to air during exfoliation and film processing [45]. Pre-adsorbed oxygen molecules take electrons from the valence band of MoS_2_, thus creating a hole accumulation region (HAR), as represented by Equation (2) and depicted in Figure 13. When NO_2_ (an electron-acceptor gas) molecules are adsorbed on the 2H-MoS_2_ surface, they act as electron acceptors (Equation (3) and Figure 13). Therefore, they attract electrons from 2H-MoS_2_, leading to an increase in hole concentration, which is consistent with an increase in the conductance, e.g., a decrease in the resistance of the MoS_2_ as the NO_2_ concentration increases.

In MoS_2_ TMDCs, defects such as S vacancies (usually denoted as V_S_) have a critical fundamental rule in the sensing mechanism toward gas molecule species contributing to carrier charge transfer. It is confirmed from previous reports that the adsorption of NO_2_ on the 2H-MoS_2_ surface is considered unfavorable without the existence of chalcogenide vacancies.

## 4. Conclusions

In summary, we have demonstrated that a high-shear mixing method produces a MoS_2_ nanosheet dispersion with a narrow sheet-size distribution, and a vacuum-assisted filtration method is able to produce compact film suitable for gas sensing, which is easy and cost-saving compared to conventional CVD techniques. The thin films with gold interdigitated electrodes were used to develop large-area chemiresistive gas sensors to detect NO_2_. The MoS_2_-based gas sensor shows a reversible response–recovery characteristic towards NO_2_ at 30 °C, but it exhibits a slow recovery due to the strong bonding between gas molecules and the MoS_2_ surface. Moreover, the responsivity to a low NO_2_ concentration of 1 ppm was demonstrated, making MoS_2_ a promising candidate for gas sensing applications. In terms of response optimization, we investigated thin-film thickness and working temperature. By studying the NO_2_ responses of 150 nm and 300 nm MoS_2_ thin films, it shows that thinner films process a higher response, which is due to a higher surface-to-volume ratio. By increasing the working temperature from 30 °C to 100 °C, we observed that the thin-film response decreased and recovery time increased with increasing temperature.

## Figures and Tables

**Table 1:** Comparison of the performance of MoS_2_ based sensors to NO_2_ gas.

Material	Fabrication Method	Type of Sensors	OT (°C)	NO_2_ Concentration (ppm)	Response (%)	Res/Rec Time (s)	Ref.
MoS_2_ TFTs	Mechanical exfoliation	FET	RT	1.2 ppm	6.1%	>30 min	[34]
4 L MoS_2_	CVD	FET	RT	10 ppm	5%	Not reported	[35]
MoS_2_ nanosheets	Liquid exfoliation + drop casting	Chemiresistor	RT	0.5 ppm	81%	~110 s/~120 s	[31]
MoS_2_/graphene	Mechanical exfoliation + CVD	Chemiresistor	100 °C	1.2 ppm	~3%	Not reported	[36]
MoS_2_/PSiNWs	Chemical etching + CVD	Chemiresistor	RT	1 ppm 5 ppm	0.27% 5.75%	−/>60 min	[37]
MoS_2_ layers	CVD	Chemiresistor	RT	1 ppm 10 ppm	0.4% 0.5%	Not reported	[38]
NbS_2_ nanosheets	CVD	Chemiresistor	RT	5 ppm	18%	3000 s/9000 s	[39]
WSe_2_ monolayer	CVD	Chemiresistor	250 °C	100 ppb	-	18 s/38 s	[40]
MoS_2_ nanosheets	Liquid exfoliation + vacuum filtration	Chemiresistor (300 nm)	30 °C	1 ppm 5 ppm	1.5% 4.5%	114 s/420 s 6 min/60 min	This work

**Table 2:** The effect of film thickness on sensitivity of the MoS_2_ nanosheet thin-film gas sensors for two different values of concentrations of NO_2_ gas.

MoS_2_ Film Thickness	NO_2_ Concentration	
	1 ppm	2 ppm
150 nm	4.5%	7%
300 nm	1.5%	1.8%

## Data Availability

Not applicable.
